# A 360-degree assessment of teaching effectiveness using a structured-videorecorded observed teaching exercise for faculty development

**DOI:** 10.1080/10872981.2019.1596708

**Published:** 2019-04-11

**Authors:** Christopher A. Jones, Franklin S. Watkins, Julie Williams, Ann Lambros, Kathryn E. Callahan, Janice Lawlor, Jeff D. Williamson, Kevin P. High, Hal H. Atkinson

**Affiliations:** a Section on Gerontology and Geriatric Medicine, Wake Forest School of Medicine, Winston-Salem, NC, USA; b Health and Exercise Science, Wake Forest University, Winston Salem, NC, USA; c Department of Internal Medicine, Section on Infectious Diseases, Vice President Health System Affairs, Wake Forest School of Medicine, Winston-Salem, NC, USA; d Department of Internal Medicine, Section on Gerontology and Geriatric Medicine, Wake Forest School of Medicine, Winston-Salem, NC, USA

**Keywords:** Microteaching, faculty development, teaching methods, 360 degree assessment, evaluation

## Abstract

**Background:** Filming teaching sessions were reported in the medical literature in the 1980s and 1990s but appear to have been an underreported and/or underutilized teaching tool since that time. National faculty development programs, such as the Harvard Macy Institute (HMI) Program for Educators in Health Professions and the Stanford Faculty Development Center for Medical Teachers program, have attempted to bridge this gap in formal instruction in teaching skills through microteaching sessions involving videos for self- and peer-assessment and feedback.

**Objective:** Current video-feedback faculty development initiatives are time intensive and impractical to implement broadly at an institutional level. Further, results of peer feedback have not been frequently reported in the literature at the institutional level. Our research aims to propose a convenient and effective process for incorporating video analysis into faculty devleopment programs.

**Design:** Our work describes a novel technique using video-recorded, simulated teaching exercises to compile multi-dimensional feedback as an aid in faculty development programs that promote teaching-skill development. This research evaluated the effectiveness of a focused teaching practicum designed for faculty in multiple specialty departments with large numbers of older patients into a geriatrics-based faculty development program. Effectiveness of the practicum is evaluated using quantitative scoring and qualitative analysis of self-reflection as well as peer and trainee input.

**Results:** VOTE sessions demonstrate an important exportable product which enable faculty to receive a detailed 360-degree assessment of their teaching.

**Conclusion:** This intervention can be easily replicated and revised, as needed, to fit into the educational curriculum at other academic medical centers.

## Introduction

Within the past generation of trainees, the culture of medical education has shifted from lecture-based didactic sessions to more interactive, patient-based learning environments. [1,2], This cultural shift presents a new challenge in teaching physicians the requisite skills to facilitate small group discussions as well as teach on clinical rotations. The changing national landscape of contemporary medicine has also compressed many patient visits in outpatient clinics and on inpatient ward services to brief encounters, highlighting the need for effective teaching skills when time for teaching is limited.

Many new physicians enter careers in academic medicine with little, if any, formal training in teaching methodology yet are expected to serve as mentors and teachers to learners at various levels of training. [3–5], Although faculty development is a requirement from the Accreditation Council of Graduate Medical Education, experienced faculty members often have minimal or no faculty development to optimize teaching methods that effectively engage adult learners. Formal feedback from colleagues regarding teaching performance may be an effective tool to improve teaching skills, but opportunities for this type of feedback are usually limited in the frequency and specificity. [6,7], Faculty development programs have been proposed as a means to identify and address these limitations. However, barriers including time constraints, faculty willingness to participate in peer evaluation, and lack of defined processes for robust evaluation may limit programs’ inclusion of specific teaching assessments [8–10]. Filming teaching sessions were reported in the medical literature in the 1980s and 1990s but appear to have been an underreported and underutilized teaching tool since that time. [11–13], National faculty development programs, such as the Harvard Macy Program for Educators in Health Professions [14] and the Stanford Faculty Development Center for Medical Teachers program [15] have attempted to bridge this gap in formal instruction in teaching skills through microteaching sessions involving videos for self- and peer-assessment and feedback. These initiatives are time intensive and impractical to implement broadly at an institutional level. Further, results of peer feedback have not been frequently reported in the literature at the institutional level.

The faculty in Gerontology and Geriatric Medicine at Wake Forest School of Medicine (WFSM) saw an opportunity to incorporate a focused teaching practicum for faculty within a multiple-specialty faculty development program. 360-degree assessments involve a combination of feedback from subordinates, colleagues and superiors. 360-degree feedback has been considered an essential tool in transformational leadership because the evaluation process avoids bias through diversity of viewpoints represented, and it is rarely applied to teaching assessments. Specifically, we designed a teaching practicum using a Videorecorded Observed Teaching Exercise (VOTE) to provide self-, peer- and learner assessments of teaching. In this article, we summarize the development, implementation and evaluation of our VOTE program in several domains: feasibility, faculty satisfaction, perception of effectiveness, and quantitative and qualitative differences in self-, peer- and learner ratings of teaching.

## Methods

### Educational setting and rationale for VOTE sessions

The Donald W. Reynolds Foundation provided funding from 2009 to 2017 to enhance the geriatrics curriculum at WFSM. One aim was to develop and implement a geriatrics-based faculty development program for specialists[16], so as to develop geriatrics education leadership outside of the Section on Gerontology and Geriatric Medicine and increase exposure of learners at all levels of training within the specialties to geriatric principles and best practices for the care of older adults – so-called ‘little g’ Geriatrics[17]. We focused our faculty development program on specialties with direct inpatient and acute care services to optimize the impact for teaching in clinical settings. These specialties included Geriatrics, General Medicine, Hematology/Oncology, Nephrology, Emergency Medicine, Pulmonary/Critical Care, Endocrinology, Rheumatology, Infectious Disease, Gastroenterology, General Surgery, Hospital Medicine, Orthopedics, and Urology.

We partnered with the University of Chicago to modify their Curriculum for the Hospitalized Aging Medical Patient (CHAMP) [18] to meet the needs of specialists at our institution. Modifications included adding case-based and interactive components which oriented participants to principles of effective small group teaching while learning geriatric content. Participants were asked to include a geriatric topic related to their area of specialty in their VOTE teaching session. The modified curriculum, Geriatrics Principles for Specialists (GPS), was a case-based, interactive program that highlights core topics in geriatrics for our specialty colleagues. A cornerstone of GPS was the use of a videorecorded teaching exercise to provide faculty with an opportunity to obtain a comprehensive assessment of teaching skills in a structured environment.

In developing VOTE, we recognized that faculty at WFSM rarely have an opportunity to observe their own teaching and infrequently observe their peers in a teaching session. In addition, faculty are infrequently provided specific feedback regarding their teaching skills from peers except for high-stakes evaluation of work performance. We hypothesized that developing a faculty development tool which provides faculty with a multidimensional assessment from a specific teaching encounter would be both feasible and acceptable to faculty. This battery of feedback from multiple perspectives would not only provide a ‘360-degree’ assessment which could be used for academic promotion, but also provide an opportunity for self-reflection on teaching.

### Design of the VOTE sessions

Prospective VOTE faculty, actively involved as clinical educators, were identified by faculty champions in each specialty group based on their interest in geriatrics. Selected faculty were then invited to participate by direct invitation from the leadership of the GPS. Participation in GPS required that each faculty member teaches on a geriatrics topic that would be pertinent to daily rounds in the respective specialty. To engage with adult learning and the clinical environment, another requirement was that the session emphasizes finite core points and last for no more than 15 min. Faculty members received instruction on delivering an effective small group presentation and were provided a brief orientation on teaching methods including case-based discussions, ‘chalk talks’, active demonstration of concepts or maneuvers, traditional lectures, and role-playing exercises. Faculty were asked to choose one teaching method to conduct a short teaching presentation, and most chose case-based discussions as the primary teaching method. An Academic Computing Technical Assistant was retained to conduct the recording functions of the session. In contrast to the Harvard Macy and Stanford faculty development programs, since we had a local learning environment to draw from, we chose to have students and trainees in attendance for these talks to allow the sessions to be as ‘real life’ as possible and to provide each faculty member with the opportunity to use active learning techniques and trainee interaction during the sessions. Each faculty participant delivered a 15-min teaching session to 2–3 medical students and 1–2 interns on our Geriatrics rotation.

**Figure 1.**
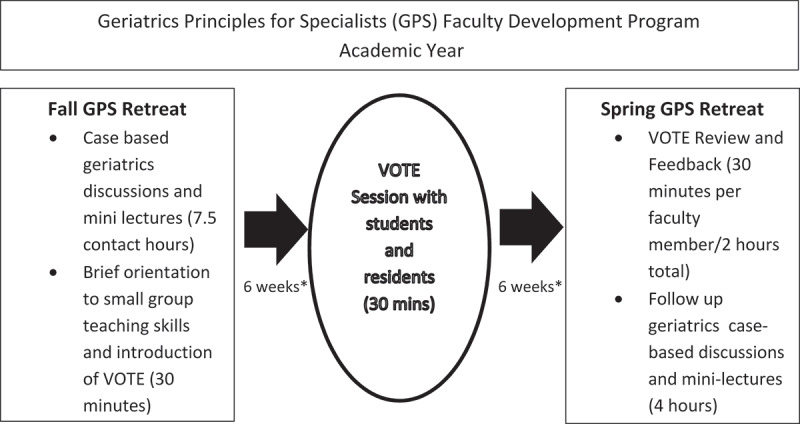
Timeline of the videorecorded observed teaching exercise (VOTE) faculty development sessions.

Approximately 6 weeks after the videorecorded teaching sessions, small groups at a GPS retreat viewed assigned VOTE recordings; each group included 2–3 peer faculty whose own recordings also were being assessed and 1–2 geriatrics faculty members who served as facilitators. This session provided the bulk of the faculty development in teaching skills for this program through direct feedback to the teaching faculty. Participants were given a brief overview on the process for generating actionable, positive feedback and providing helpful summaries of these findings to the teaching subjects. Feedback forms were provided to faculty with instructions to complete. Group consensus of the feedback to be delivered to each peer teaching subject was generated through the discussion process. A total of 30 min was spent in viewing, evaluating and providing feedback on each video. The video itself was viewed for a maximum of 15 min. After viewing the video, the faculty member whose session was being assessed exited the room to perform a self-evaluation of teaching while peers completed peer-assessment forms. Next, peers spent approximately 5 min discussing the teaching session and developing specific group feedback for their colleague. For the final 10 min of each session, the subject faculty member returned and summarized his/her personal assessment of the teaching, including effective methods and areas for improvement. Peers and geriatrics faculty then provided group feedback. See timeline in Figure 1. In total, faculty participants allocated approximately 6 h to the activity including preparation for the session, performing the recording, viewing, debriefing, then returning for a follow up session to share feedback.

### Evaluation

At the end of each teaching session, medical students and interns serving as the trainee audience for the session completed an anonymous feedback survey, developed by a PhD educator on the study team. The survey presented 20 statements which describe teaching quality, assessing clarity of presentation, organization, engagement of the instructor, approachability of the instructor, and interactivity of the session. At the end of each survey, open-ended questions prompted the rater to identify both the most effective teaching strategies implemented by the faculty member and areas where the teacher could improve. The same survey was used at the faculty retreat during the observation of each VOTE session by the faculty members being evaluated and their peers in the small group sessions. To promote candid feedback, peer survey feedback when presented to the subject faculty member was anonymous. Student and intern feedback gathered previously was anonymous and aggregated and presented to the subject faculty member at this time as well.

For the 2009–2013 cohort (n = 24) an additional evaluation was requested within one week of completion of the GPS retreat. Faculty subjects were asked to reflect on the value and effectiveness of using the sessions as a faculty development vehicle. This anonymous survey consisted of 11 statements rated on a 5-point Likert scale (5 = strongly agree, 1 = strongly disagree). Survey items addressed the value of various aspects of the VOTE experience, faculty comfort in receiving and providing feedback to peers, plans to incorporate new content and teaching techniques from the experience, and overall change in confidence in teaching.

All evaluative tools and reporting of results for our Reynolds Foundation program initiatives were approved by the WFSM Institutional Review Board.

### Analysis

A total mean teaching score for each trainee-, peer-, and self-evaluation survey was computed combining the average ratings of the 20 survey items. These scores were compared among the three groups in pairwise comparisons using t-tests to determine quantitative differences. An applied thematic analysis was used to analyze qualitative comments from the survey data to pinpoint themes presented by multiple evaluators. To enumerate the variety of themes and patterns occurring within and across the data set, we drew on guidance offered by Braun and Clarke (2006). Specifically, they advise that it is up to the researcher(s) to decide what counts as a theme, and that this should be done using a flexible process without applying ‘rigid rules’. [19,20] Open-ended feedback comments from the trainee-, peer-, and self-assessments were reviewed and compared for differences in themes. Together, these authors (J. L., F. W., J.W.) developed categories and tested a coding scheme based on review of the comments and coding of sample text. Interpretations were made together. An audit trail was kept, and discrepancies were resolved through consensus. These authors concurred on the final themes as reported in the results. In addition, post-retreat faculty satisfaction survey results are presented as proportions at each level of agreement with each statement on the 5-item Likert scale.

## Results

**Figure 2.**
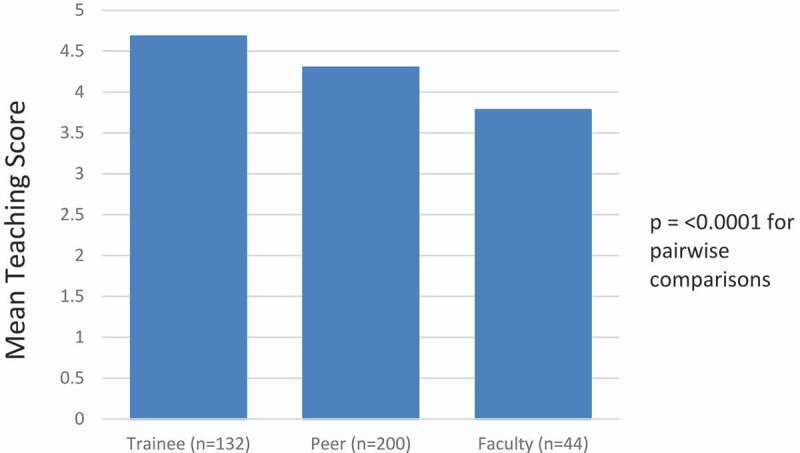
Comparison of trainee, peer and self-ratings of teaching.

Throughout GPS, a total of 49 faculty members from 14 specialties completed the VOTE program (Table 1). Results from trainee-, peer- and self-assessments of the VOTE session are provided in Figure 2. Trainees rated teaching the highest among the three groups, with a mean score of 4.68. Faculty peers rated teaching slightly lower at a mean of 4.30, and self-assessments were the lowest mean score at 3.78. All differences between the three comparison groups (self and peer, self and trainee, and peer and trainee) achieved statistical significance (p = <0.0001).

**Table 1.** Faculty participants (*n* = 49) in Geriatrics Principles for Specialists (GPS).

Specialties	Number of participants
Geriatrics	6
General medicine	8
Hematology/oncology	7
Nephrology	2
Emergency medicine	7
Pulmonary/critical care	6
Endocrinology	1
Rheumatology	2
Infectious disease	1
Gastroenterology	1
General durgery	2
Hospital medicine	3
Orthopedics	2
Urology	1

On average, the number of feedback comments available to participating faculty was as follows: strengths: 10 comments from peers and 4 comments from trainees; weaknesses: 6 comments from peers and 1 comment from trainees. Qualitative analysis of faculty and trainee participant feedback yielded four specific thematic domains of responses about the teachers: 1) communication skills (verbal and non-verbal); 2) interactivity of the session and engagement with learners; 3) materials used for teaching (relevance, clear objectives, amount of content), and 4) teaching methods, including supportive materials and references. Table 2 reports areas of strength and potential areas of improvement for faculty subjects measured by volume of themed responses reported by trainee-, peer- and self-assessments. Themes of interactivity and teaching method were most frequently reported by all subjects as perceived strengths. Faculty responses, which indicated areas for improvement, were most-frequently themed: communication skills (41%) and materials used for teaching (27%). Peers most-frequently reported areas for improvement themed: materials used for teaching (31%) and communication skills (29%). Trainees perceived areas for improvement in themes: materials used for teaching (32%) and teaching method (33%).

**Table 2.** Qualitative themes of responses from trainee-, peer- and self-assessments of VOTE sessions.

Number of responses			
Thematic areas of strength	Trainee (*n* = 170)	Peer (*n* = 428)	Self (*n* = 58)
Communication skills	19 (11%)	91 (21%)	7 (12%)
Materials used for teaching	26 (15%)	101 (24%)	11 (19%)
Interactivity	68 (40%)	132 (31%)	27 (47%)
Teaching method	57 (34%)	104 (24%)	13 (22%)
Thematic areas of weakness	Trainee (*n* = 40)	Peer (*n* = 251)	Self (*n* = 73)
Communication skills	5 (12%)	72 (29%)	30 (41%)
Materials used for teaching	13 (32%)	77 (30%)	20 (27%)
Interactivity	9 (23%)	65 (26%)	16 (22%)
Teaching method	13 (33%)	37 (15%)	7 (10%)

Of the 49 faculty members to complete taping of VOTE sessions, faculty from the early GPS cohort (n = 38) received a post-retreat self-reflection survey. Sixty-three percent of that cohort returned the completed survey. Of those respondents, all agreed (29.2%) or strongly agreed (70.8%) that both the session itself and subsequent self-reflection about their teaching was a valuable exercise to improve their teaching skills. In addition, all agreed (37.5%) or strongly agreed (62.5%) that watching the teaching sessions of their peers provided them with new techniques for their teaching. Only a minority of respondents reported that delivering (12.5%) or receiving (20.8%) peer feedback was uncomfortable.

## Discussion

Our design of videorecorded microteaching sessions embedded into a faculty development program presents a feasible, well-received model to provide faculty development in teaching and a robust 360-degree assessment of teaching skills. Our results demonstrated that faculty were receptive to feedback from others regarding their teaching and identified new teaching skills through these sessions that they planned to incorporate into their future teaching. Overall assessment of teaching skills was quantitatively and qualitatively different among trainee-, peer- and self-assessments. This suggests, along with the faculty satisfaction data, that adding peer- and self-assessments to the standard faculty assessment provides additional valuable information for the teacher, consistent with our hypothesis. We estimated that faculty would have lower ratings when they actually observed themselves, and also that they would probably focus more on things that others may be more uncomfortable in pointing out such as distracting mannerisms, clarity of language, etc. This was confirmed in our analyses. The area that faculty focused on for improvement in their self-assessment most frequently was communication skills. Neither giving nor receiving feedback from peers was uncomfortable for the majority of participants.

Two strengths of our program are that it is feasible and reproducible. While the session and feedback were embedded within a faculty development program for geriatrics content, the current model of our VOTE sessions would work equally as a ‘stand alone’ product that could be used for new faculty or any section of faculty interested in evaluating their own teaching within a peer group.

In addition, costs for these sessions were low. VOTE video capture costs ranged from $45 – $90 per session depending on the audiovisual capacity of the room used for recording. Costs for this activity included an audiovisual technician who performed the room setup and videorecording. However, a handheld videorecorder or mobile device could be used for these sessions as well. Other resources required for implementation of the VOTE activity within an academic medical center include an administrator with time allocated to schedule and coordinate the VOTE sessions. It is also helpful, but not required, to make this a part of an existing faculty development course for medical educators in which to promote this activity.

Another strength of the program was the participation of residents and students at WFSM as the learner audience for the teaching practicum. When initially developing the VOTE session concept, we considered using the faculty role-play model, but feedback from colleagues included concern that the sessions would seem artificial and not representative of the ‘real world’ of academic medicine. Including trainees, however, allowed for the inclusion of many active learning techniques as well as an audience ready to learn about geriatrics topics. An additional opportunity for implementation of VOTE sessions is in resident and fellowship education as a method to obtain a 360-degree assessment of resident or fellow teaching skills.

Limitations include the response rate to our voluntary faculty satisfaction survey. While a 63% response rate is not unexpected for physicians in a voluntary survey, [21,22], the response rate may signal underrepresentation of participants who might have felt uncomfortable either receiving or providing feedback, although anonymity was assured. Also, self-reflection data are only available for the initial cohort.

Another possible limitation of the program is participants in the faculty development program agreed to participate in the program knowing initially that VOTE microteaching sessions would be a component of the program. This volunteerism may not be seen in faculty who might be more resistant to peer and trainee feedback, which could potentially limit its effectiveness if used on a mandatory basis for teaching evaluation. Finally, dedicated time to both record and evaluate the videos is required to optimally implement the program. While we were fortunate to have grant funding to assist in this effort, not all institutions may be able to readily incorporate this modality.

In conclusion, our VOTE ‘microteaching’ sessions provided robust feedback to the individual participants, including 360-degree teaching assessments; they were well-received and rated highly by participating faculty members. Most participants returning post-retreat surveys reported learning new teaching techniques to incorporate in their future teaching and felt the sessions enhanced their teaching. In some settings, such as assessment of competence in patient care, physicians have a limited ability to self-assess[23]. However, we are unaware of any studies that have directly compared the differences in self-assessment of teaching using videorecordings to trainee and peer assessments. Our analyses underscore that there is value in peer and self-assessment using videorecording because in the current medical education system, most evaluations are by provided by trainees who may base ratings on personality of the faculty. Peers may be more objective in providing feedback and based in professional perspective. Self-evaluations may be more critical overall, but they focus on more on communication skills than others as areas to improve. We think that the use of video is a major benefit to the program and actually yields different results than other programs that have compared self-assessment to learner assessment. For example, a recent study of an Observed Structured Teaching Encounter aimed at improving attending physician communication skills, attending physicians actually rated their skills higher than learners, contrary to what we observed [24] ^–^ we think that a major difference is the use of video for self-assessment. By providing the variety of perspectives, self, peer and trainee, the VOTE method offers a balanced view of faculty teaching. Furthermore, the targeted, directed teaching sessions mimicked the teaching that organically occurs on busy clinical teaching services. Additionally, the structure for VOTE sessions could fit within future faculty development programs or be carved out as an independent method to provide educators with a 360-degree assessment. Thus, we view our VOTE sessions as a useful tool for improving faculty development at academic medical centers through detailed 360-degree assessments of their teaching methods. The VOTE method can be replicated and revised, as needed, to fit into the educational curriculum at other academic medical centers.
